# A lightweight piecewise linear synthesis method for standard 12-lead ECG signals based on adaptive region segmentation

**DOI:** 10.1371/journal.pone.0206170

**Published:** 2018-10-19

**Authors:** Huaiyu Zhu, Yun Pan, Kwang-Ting Cheng, Ruohong Huan

**Affiliations:** 1 College of Information Science and Electronic Engineering, Zhejiang University, Hangzhou, Zhejiang, China; 2 Department of Electronic & Computer Engineering, Hong Kong University of Science and Technology, Hong Kong, China; 3 College of Computer Science and Technology, Zhejiang University of Technology, Hangzhou, Zhejiang, China; University of Auckland, NEW ZEALAND

## Abstract

This paper presents a lightweight synthesis algorithm, named *adaptive region segmentation based piecewise linear* (ARSPL) algorithm, for reconstructing standard 12-lead electrocardiogram (ECG) signals from a 3-lead subset (I, II and V2). Such a lightweight algorithm is particularly suitable for healthcare mobile devices with limited resources for computing, communication and data storage. After detection of R-peaks, the ECGs are segmented by cardiac cycles. Each cycle is further divided into four regions according to different cardiac electrical activity stages. A personalized linear regression algorithm is then applied to these regions respectively for improved ECG synthesis. The proposed ARSPL method has been tested on 39 subjects randomly selected from the PTB diagnostic ECG database and achieved accurate synthesis of remaining leads with an average correlation coefficient of 0.947, an average root-mean-square error of 55.4μV, and an average runtime performance of 114ms. Overall, these results are significantly better than those of the common linear regression method, the back propagation (BP) neural network and the BP optimized using the genetic algorithm. We have also used the reconstructed ECG signals to evaluate the denivelation of ST segment, which is a potential symptom of intrinsic myocardial disease. After ARSPL, only 10.71% of the synthesized ECG cycles are with a ST-level synthesis error larger than 0.1mV, which is also better than those of the three above-mentioned methods.

## Introduction

The standard 12-lead electrocardiogram (ECG) is routinely used to screen for heart disease. The procedure is usually performed in a medical institution; self-care and home use are not possible. However, acute heart abnormalities, which can be easily missed on routine ECG examination, are associated with high risks of heart failure. Hence, ECG monitoring in everyday life would be useful to prevent emergencies in the elderly and those with recognized heart disease [1].

Traditional Holter monitors measure the standard 12-lead ECG continuously for more than a day, yet they are inconvenient to carry and limiting usual activities such as bathing and arm-waving. With recent advances in mobile computing and wearable technology, daily ECG monitoring and signal analysis have become feasible. However, almost all wearable ECG monitoring devices provide only a limited number of ECG leads to allow portability and comfort [2]. Cardiologists generally follow the standard 12-lead ECG concept and have been trained to interpret heart conditions using 12-lead systems. Therefore, it is essential to synthesize standard 12-lead ECGs using signals from a reduced number of leads [3]. Considerable correlations are evident among the signals of various leads of a standard 12-lead system [4], rendering standard 12-lead ECG synthesis from a subset of leads feasible.

The heart–torso electrical system could be considered as linear and quasi-static, since the tissue capacitance and the electromagnetic propagation effect could be neglected [3]. Therefore, it is justifiable to consider the human body as a homogeneous and heart-centered sphere, leads of the standard 12-lead ECG system can be mapped as lead vectors distributed in the frontal and transverse planes [4], thus a common linear regression (LR) method could be applied to reconstruct the full 12-lead ECG set from a reduced subset of leads effectively with high fidelity [5]. Moreover, nonlinear methods such as the back propagation (BP) neural network could be used for improving ECG synthesis accuracy [6] at the cost of greater computational complexity. Besides, as the form of an individual's ECG would change over time and vary with her/his body condition, the reconstruction model for synthesis needs to be updated regularly.

Continuous remote ECG monitoring requires both accurate capture of ECG signals and also an immediate response to unexpected cardiac abnormalities [7]. Therefore, to render synthesis of a standard 12-lead ECG applicable at home, accurate and efficient ECG reconstruction are critical. However, the accuracy of the common LR method is limited by the instability and discontinuity of captured ECG signals, whereas the BP method is complex, requiring extensive training and significant computing resources.

According to lead theory [8], the voltage of a given lead can be expressed as the scalar product of the lead vector $\vec{L}$ and the heart vector $\vec{p}$, where $\vec{L}$ is a vector in space describing the direction in which a certain lead monitors cardiac electrical activity (CEA) and $\vec{p}$ is a dipole describing the CEA [9]. As the source location of CEA varies during the different stages of a cardiac cycle, e.g., atrial systole (represented by P wave), ventricular systole (represented by QRS wave), ventricular diastole (represented by T wave), etc. [10], the heart vector $\vec{p}$ can be described as a single moving dipole with a time-dependent location at different stages [11]. Therefore, comparing to the method using complete ECG signals, segmentation of the ECG into regions matched to the CEA stages of the cardiac cycle, followed by modeling of these ECG regions, would better consider the differences among ECG regions at different CEA stages, enhancing ECG synthesis.

Here, we present a novel, lightweight synthetic method, which we term the adaptive region segmentation-based piecewise linear (ARSPL) method, to reconstruct the standard 12-lead ECG from three leads: I, II and V2. ARSPL enhances the accuracy of LR synthesis by applying LR to segmented ECGs rather than complete signals. The ECG is first divided into three regions representing the different CEA stages. These regions, as well as a self-defined head/tail part of the ECG signal, are then used to build separate LR models between the leads of the reduced lead set and the remaining leads of the 12-lead ECG, allowing more precise ECG synthesis.

As the ST segment of ECG is essential for diagnosing myocardial ischemia, which is a significant public heart disease [12], we define the ST-level synthesis error (STSE) as:

$$STSE={ST}_{syn}-{ST}_{ori}$$ (1)

where *ST*_*syn*_ and *ST*_*ori*_ are the ST-level of the synthesized and original ECG, respectively. The ST-level is measured in various ways in clinical practice [13], here, the ST-level is measured 60 ms after the J-point and compared with the PR segment, as commonly recommended [14]. Meanwhile, since the baseline noise could affect the ST-level dramatically, the STSE should be evaluated after the removal of baseline wandering [12]. Based on (1), we introduce a metric, *the critical denivelation ratio (CDR) of the ST-level* as:

$$CDR=\frac{\mathrm{number}\;\mathrm{of}\;\mathrm{the}\;\mathrm{cardiac}\;\mathrm{cycles}\;\mathrm{with}\;|STSE\mathrm{|>}0.1mV}{\mathrm{number}\;\mathrm{of}\;\mathrm{all}\;\mathrm{cardiac}\;\mathrm{cycles}}$$ (2)

to evaluate denivelation caused by synthesis to this important parameter of the original ECGs. The CDR can be further subdivided into the elevation ratio (ER) and the depression ratio (DR) as expressed in (3)–(4).

$$ER=\frac{\mathrm{number}\;\mathrm{of}\;\mathrm{the}\;\mathrm{cardiac}\;\mathrm{cycles}\;\mathrm{with}\;STSE>0.1mV}{\mathrm{number}\;\mathrm{of}\;\mathrm{all}\;\mathrm{cardiac}\;\mathrm{cycles}}$$ (3)

$$DR=\frac{\mathrm{number}\;\mathrm{of}\;\mathrm{the}\;\mathrm{cardiac}\;\mathrm{cycles}\;\mathrm{with}\;STSE<-0.1mV}{\mathrm{number}\;\mathrm{of}\;\mathrm{all}\;\mathrm{cardiac}\;\mathrm{cycles}}$$ (4)

The proposed ARSPL method, as well as the common LR method, the BP method, and the BP method optimized with a genetic algorithm (GA-BP) have been evaluated using 39 subjects with different health conditions from the PTB diagnostic ECG database [15]. We compare the four methods based on several common metrics, such as the correlation coefficient (CC), the root-mean-square error (RMSE) and the ST-level CDR, as well as their runtime performance.

## Related work

A standard 12-lead ECG set consists of three limb leads (I, II, III), three augmented limb leads (aVR, aVL, aVF) and six precordial leads (V1 to V6). The augmented limb leads can directly be calculated from any two of the limb leads based on (5)–(8).

I–II–III=0 (5)

aVR=−(I+II)/2 (6)

aVL=I–II/2 (7)

aVF=II–I/2 (8)

Considering the arithmetic relationship above and the orthogonal relationship [3] of a standard 12-lead ECG, we choose I, II and V2 as a 3-lead subset to reconstruct its standard 12-lead ECG. Since the lead III and the augmented limb leads can be directly derived from the 3-lead subset, the main problem is the reconstruction of the remaining five precordial leads (V1, V3, V4, V5, and V6) from the 3-lead subset. In this section, we review two leading synthesis methods as follows.

### Linear regression

The linear regression method is the most commonly used ECG reconstruction method. Dower [16] first introduced the LR method for 12-lead ECG reconstruction from the Frank system for which the transformation matrices were calculated based on the Frank image surface [17]. Uijen et al. [18] later demonstrated that the transformation matrices generated by the universal LR method performs better than Dower’s matrices for 12-lead ECG synthesis from the Frank system. Nelwan et al. [5] concluded that the precordial leads in a 12-lead system have sufficient correlation among them and a missing precordial lead can be well reconstructed from the remaining precordial leads using the LR method. The authors of [5] also showed that the personalized LR synthesis method generally achieves a better CC than the universal LR method. The authors of [19–21] applied the LR method for 12-lead ECG synthesis from specialized leads in other lead systems, i.e., the “Transtelephonic System”, the “Eigenleads System” and the “Trobec and Tomašić System”.

In our case, 3 leads are selected as the initial subset *T* for ECG reconstruction, where *T* can be expressed as:

T={I,II,V2} (9)

The standard 12-lead ECG system is denoted as:

R={I,II,III,aVR,aVL,aVF,V1,V2,V3,V4,V5,V6} (10)

For a lead *L* ∈ *R*, *L* can be reconstructed from *T* by the LR method:

$$L=a_{L}+b_{L}∙I+c_{L}∙II+d_{L}∙V2$$ (11)

where (*a*_*L*_,*b*_*L*_,*c*_*L*_,*d*_*L*_) is the linear coefficient set between lead *L* and set *T*.

When there are *N* sets of samples, denoted as (*T*_1_,*R*_1_),…,(*T*_*i*_,*R*_*i*_),…,(*T*_*N*_,*R*_*N*_), where *N* > 3, the linear model between *R* and *T* can be expressed in the following matrix form:

R=X·β+ε (12)

where ***R*** is an *N* × 12 matrix consisting of row vectors of samples *R*_*i*_, ***X*** is an *N* × 4 matrix containing row vectors of samples *T*_*i*_ which can be expressed as:

$$X=\left[\begin{array}{cc} 1 & T_{1}\\ \vdots & \vdots \\ 1 & T_{N} \end{array}\right]$$ (13)

***β*** is a 4 × 12 transformation matrix consisting of column vectors of linear coefficient sets (*a*,*b*,*c*,*d*) between the twelve standard leads *R* and *T*, and ***ε*** is an *N* × 12 error matrix.

Reconstructing the standard 12-lead ECG requires calculation of the estimated matrix ***b*** of the conversion matrix ***β***. Based on the least-square criterion,

$$b={\left(X^{T}X\right)}^{-1}X^{T}R$$ (14)

The resulting matrix $\hat{R}$ of the standard 12-lead ECG synthesis can then be calculated as:

$$\hat{R}=X\cdot b$$ (15)

### Back propagation neural network

The back propagation (BP) neural network is a non-linear approach for ECG synthesis which involves the following steps:

1. Establish an initial BP neural network with random weights and thresholds;
2. Use the ECG subset S, for example, I, II and V2 as the input to the network, and the expected subset U, in this case, V1 and V3 to V6 as the output of the network for the training process;
3. After training, the ECG synthesis BP neural network is used to reconstruct the signals of the expected ECG leads.

The initial weights and thresholds affect the performance of the BP neural network method significantly. To overcome this limitation, Atoui et al. [6] proposed a method to train multiple BP neural networks using the same data set and the final synthesis result is the average of the ECG sequences reconstructed from each neural network.

Another solution is to find a set of initial weights and thresholds to ensure high quality results. Instead of using random initial weights and thresholds, Chen et al. [22] proposed a GA-BP method which uses the genetic algorithm to find an optimized set of initial parameters of the BP neural network, followed by the standard training process of the BP neural network.

## Proposed ARSPL method

The moving dipole model indicated that the position of the heart vector $\vec{p}$ changes with different CEA stages. Therefore, a piecewise linear model would better describe the relationships among the standard 12-lead ECG sequences than the common LR method. We segmented the ECG sequence into regions associated with different CEA stages and built ECG subsequences by juxtaposing regions belonging to the same CEA stages. We then performed separate LR for each subsequence and used the LR models to synthesize signals for the different ECG regions.

To render segmentation simple, ARSPL performs R-peak based ECG segmentation, rather than accurate ECG sub-wave extraction. Specifically, instead of using multiple feature points, the segmentation process of the proposed method requires only adaptive detection of R-peak positions. It then utilizes a set of Experienced Time Windows for automatic segmentation of ECGs with high efficiency.

**Fig 1:**
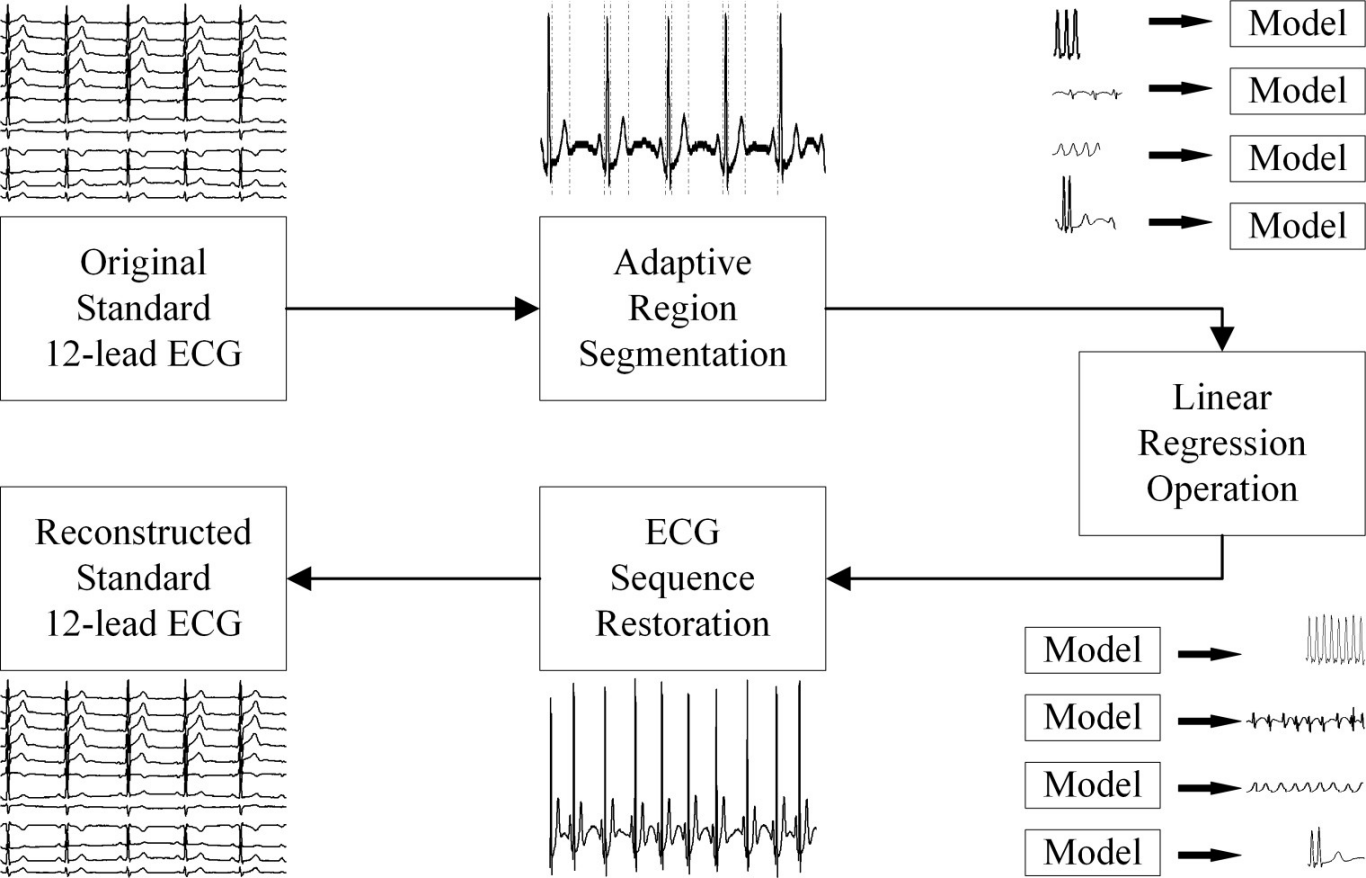
Block diagram of ARSPL.

The proposed ARSPL method, illustrated in Fig 1, consists of adaptive region segmentation, linear regression operation and ECG sequence restoration.

### Adaptive region segmentation

ECG signals are automatically divided into four regions reflecting the different CEA stages: the ST-T region, the resting-P (R-P) region, the QRS region, and the head–tail (H-T) region. The ST-T region includes the ST segment and the T wave that follow the QRS wave, principally representative of ventricular diastole. The R-P region consists of the resting phase and the P-wave between adjacent cardiac cycles, corresponding to atrial systole. The QRS region contains the complex QRS wave, reflecting ventricular systole. The H-T region is defined as the part by juxtaposing the segment prior to the end of the S wave of the first cardiac cycle with the segment after the start of the last cardiac cycle’s Q wave, thus basically the beginning and end of an ECG sequence. Although the ECG start and end points are random, dividing the H-T region ensures unique ECG segmentation pattern, which will be explained later in this section.

**Fig 2:**
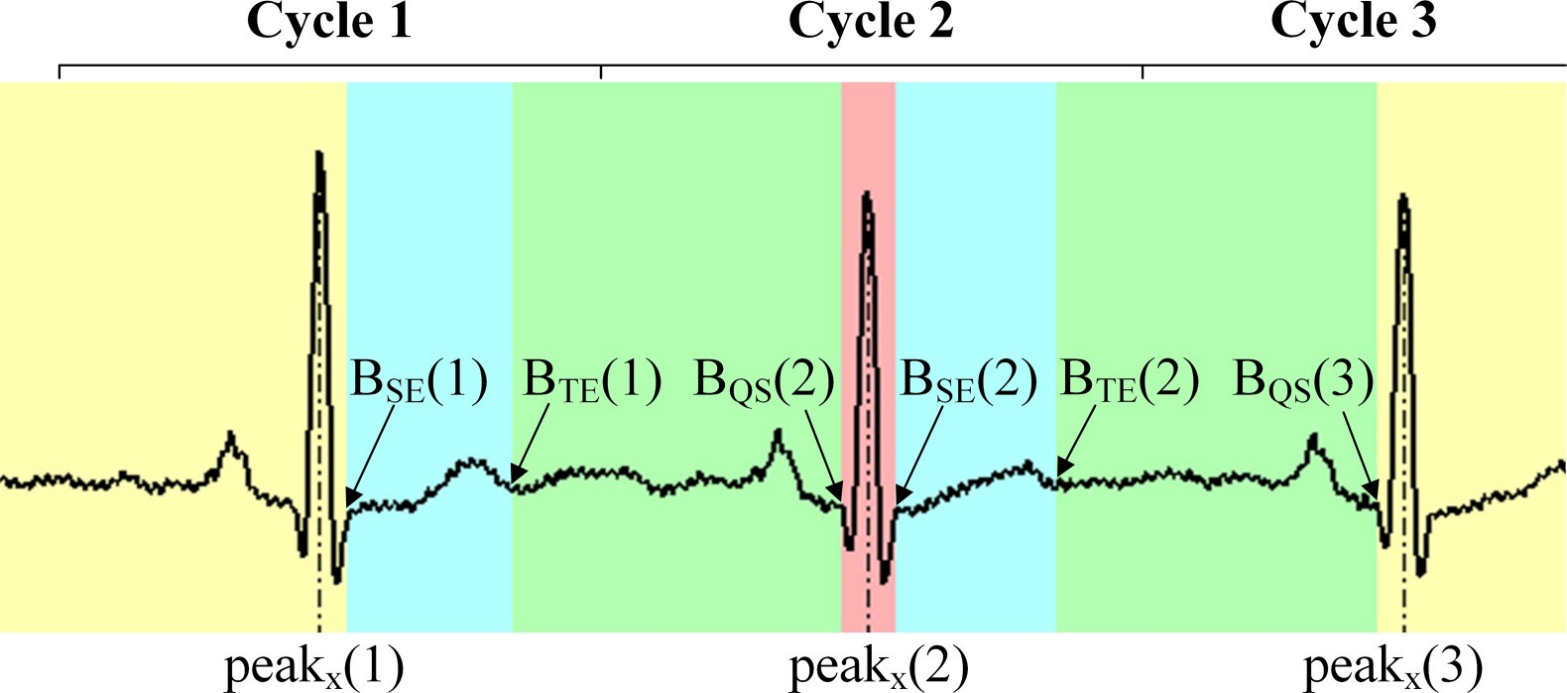
Different regions and boundaries of an ECG sequence with three R peaks (Data: s0459_re of patient233 from the PTB diagnostic ECG database). The blue regions indicate the ST-T region, the green regions indicate the R-P region, the red region indicates the QRS region, and the yellow regions indicate the H-T region.

To partition the original ECG sequence into the four regions mentioned, three boundaries, i.e., the end of the S wave (*B*_*SE*_), the end of the T wave (*B*_*TE*_), and the start of the Q wave (*B*_*QS*_) have to be determined. For an ECG sequence with *M* R peaks, we denote the cardiac cycles as *Cycle* 1 to *Cycle M*, each of which includes an R peak at *peak*_*x*_(*n*),*n* = 1,…,*M*, and we assume *M* ≥ 3. Note that an ECG sequence may start at any point of *Cycle* 1 and, similarly, may end at any point of *Cycle M*. In *Cycle* 1, we can always find the end of the S wave and the end of the T wave; however, the start of the Q wave may be absent if the QRS wave is incomplete over the cycle. On the other hand, in *Cycle M*, we can always find the start of the Q wave, yet the end of the S wave and the end of the T wave may be absent. Hence, regardless of the start point of the first cycle or the end point of the last cycle, we extract the end of the S wave and the end of the T wave only from *Cycle* 1 to *Cycle M* − 1 to get *B*_*SE*_(*n*) and *B*_*TE*_(*n*), *n* = 1,…,*M* − 1, and we extract the start of the Q wave only from *Cycle* 2 to *Cycle M* to obtain *B*_*QS*_(*n*), *n* = 2,…,*M*. An example of the regions and boundaries of an ECG sequence with three R peaks is shown in Fig 2.

Generally, to identify the boundaries of ECG sub-waves in an ECG sequence, all major feature points, including the P-peak, R-peak, Q/S-valley and T-peak points, have to be extracted. Furthermore, there are different feature-specific extraction conditions to be met, and multiple search processes are needed as the extracted information of some key feature points, like the R-peak point, is a prerequisite of correct extraction of other feature points, and in turn for high precision of ECG region segmentation. However, such a complex process incurs significant computational complexity, and thus less feasible for implementation in wearable devices. Therefore, we propose a lightweight algorithm for boundary determination in the ARSPL framework, which will be described in the next section.

Once the boundaries are determined, the ECG sequences are divided into four regions: The ST-T region of *Cycle n*, i.e., the *ST*-*T*(*n*), consists of the point index ranges from *B*_*SE*_(*n*) + 1 to *B*_*TE*_(*n*),*n* = 1,…,*M* − 1; the R-P region of *Cycle n*, i.e., the *R*-*P*(*n*), consists of the point index ranges from *B*_*TE*_(*n* −1) + 1 to *B*_*QS*_(*n*), *n* = 2,…,*M*; the QRS region of *Cycle n*, i.e., the *QRS*(*n*), consists of the point index ranges from *B*_*QS*_(*n*) + 1 to *B*_*SE*_(*n*),*n* = 2,…,*M* − 1; and the H-T region’s head part contains the ECG fragment from the beginning to the point index *B*_*SE*_(1), and its tail part contains the ECG fragment after the point index *B*_*QS*_(*M*).

**Fig 3:**
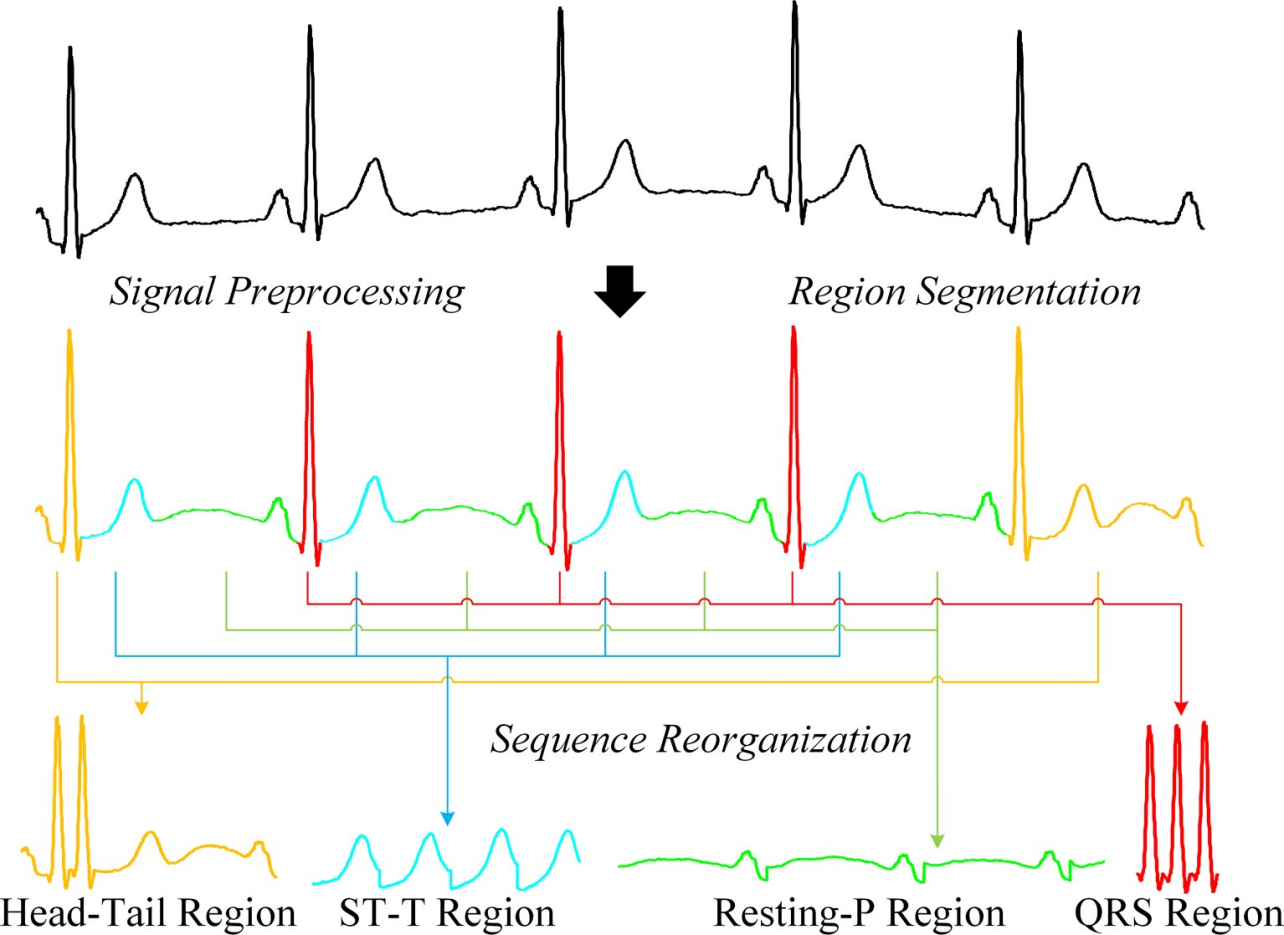
Result pattern of the ECG segmentation.

As mentioned above, the H-T region is designed to render the segmentation pattern irrelevant in terms of the start/end point of ECG signals. As the existence of *B*_*QS*_(1), *B*_*SE*_(*M*) and *B*_*TE*_(*M*) cannot be assured, as explained above, the first and the last boundary extracted are always *B*_*SE*_(1) and *B*_*QS*_(*M*). On defining the H-T region, the ECG segmentation pattern becomes fixed, as shown in Fig 3. In other words, once the R peaks are extracted, the number of each type of region is determined. This unique pattern renders the various possible segmentation situations caused by start/end point randomness irrelevant. Additionally, such segmentation is amenable to memory pre-allocation, which is important in terms of optimization [23].

#### Signal preprocessing

To improve the accuracy of ECG feature recognition, preprocessing of raw ECG signals is required before region segmentation. The types of noise having major impact on ECG signals include the baseline noise, the power line noise, and the electromyography (EMG) noise. We choose the wavelet filtering method to preprocess the ECG signals, since it has been proven to be the optimal method for ECG detrending without compromising the ST-level [24]. As described in [25], we use N-layer wavelet decomposition to remove these noises. By setting the level N approximation coefficients to zero, baseline and other low-frequency noises are largely suppressed. Furthermore, wideband noise can be eliminated by thresholding detail coefficients of each level using a positive threshold *T*_*j*_, which refers to the noise level of the *j*^*th*^ level detail coefficient.

#### Region segmentation

Identification of region boundaries relies on the positions of the R peaks, which are extracted by wavelet synthesis, adaptive amplitude thresholding and interval time window allocation as summarized in Algorithm 1.

**Algorithm 1: R Peak Determination**

**Symbol setting:**

***y*(*i*)**: ECG signal sequence

***r*(*i*)**: sequence constructed for R peak detection

***d*(*i*)**: first derivative of ***r*(*i*)**

**sign()**: sign function

***peak***_***x***_**(*n*)**: position of the n^th^ peak

***peak***_***y***_**(*n*)**: amplitude of the n^th^ peak

***N***: length of ECG sequence

***M***: total number of R peaks

**Process:**

A. Build the ***r*(*i*)** sequence from ***y*(*i*)**

        Step 1: Apply wavelet decomposition on ***y*(*i*)** and get detail coefficients.

        Step 2: Reconstruct ***r*(*i*)** from detail coefficients of the selected levels

        Step 3: Apply square operation and sliding window smoothing on ***r*(*i*)**

B. R peak detection using ***r*(*i*)**

        Step 4: Calculate the first derivative sequence ***d*(*i*)**

        Step 5: Find inflection points exceeding the threshold from ***r*(*i*)**:

        **For *i* = 1:*N***

                **if sign(d(i)) > sign(d(i + 1))&& r(i) > *threshold***

                        Register the max value within a window around ***i*** as ***peak***_***y***_**(*n*)**;

                        Register its index as ***peak***_***x***_**(*n*)**;

                **end if**

                        Value the most recent RR interval to confirm this peak

                        Adjust the amplitude threshold through recent few ***peak***_***y***_

            **end for**

C. Peak position correction

        Step 6: Adjust peak position using ***y*(*i*)**

        **for *n* = 1:*M***

                        Get few points of ***y(i)*** around ***peak***_***x***_**(*n*)**

                        Find the max value of them and get the shift

                        Peak re-registration after correction

                **end for**

As the energy of the QRS wave is distributed principally in the frequency range of 3Hz to 40Hz [26], based on the method in [27], the R peaks can be enhanced via wavelet filtering by reconstructing an R-peak detection sequence from selected scales of the wavelet detail coefficients corresponding to this frequency range.

We further proposed an improved adaptive method to extract the R peaks from the detection sequence. Specifically, the R-peak detection sequence is applied by a square operation and smoothed by a sliding window to improve the performance of R peak detection. The peak points of the detection sequence are further identified by a threshold adaptive to the recent few R-peaks’ amplitude, and the position of an incoming peak is validated by determining whether the instantaneous heart rate calculated from the interval between the two most recent peaks exceeds the common maximum heart rate of 220 bpm [28]. The sliding window smoothing in Step 3 of Algorithm 1 might introduce minor peak shifts in ***r*(*i*)** comparing to the original ***y*(*i*)**. Hence, following Step 6 of Algorithm 1, before the final registration of R peak positions, we should adjust the peak positions extracted from ***r*(*i*)** according to ***y*(*i*)** to remove peak shifts.

After the R peaks are identified, the boundaries of each region of a single ECG sequence are located using Algorithm 2. The end of the S wave (*B*_*SE*_), the end of the T wave (*B*_*TE*_), and the start of the Q wave (*B*_*QS*_) are identified using the Experienced Time Window method shown in Algorithm 2 without the need of accurate detection of sub-wave boundary of ECG sequence which effectively reduces the algorithm complexity. The experienced time window of the R-T interval, i.e., ***T***_***RT***_, is adapted to the R-peak to R-peak (R-R) intervals, since different from ***T***_***QR***_ and ***T***_***RS***_, the length of the ECG cycle affects ***T***_***RT***_ [29].

**Algorithm 2: Region Boundary Determination**

**Symbol setting:**

***SR***: sample rate of ECG signals

***peak***_***x***_**(*n*)**: position of the n^th^ peak

***rr*(*n*)**: R-R interval of the n^th^ and the n+1^th^ peak

***M***: total number of R peaks

***T***_***QR***_: experienced time window of Q-R interval

***T***_***RS***_: experienced time window of R-S interval

***T***_***RT***_: experienced time window of R-T interval

***a***_***RT***_: adjustment coefficient of R-T interval

**Process**:

A. Experienced Time Window Adjustment

        **for *n* − 1:*M* − 1**

            ***rr*(*n*) = (*peak***_***x***_**(*n* + 1) − *peak***_***x***_**(*n*))/*SR***

            ***T***_***RT***_**(n) = *a***_***RT***_ * *rr*(*n*)

        **end for**

B. Process The First/Last Cycle

        ***B***_***SE***_**(1) = *peak***_***x***_**(1) + *T***_***RS***_ * *SR*

        ***B***_***TE***_**(1) = *peak***_***x***_**(1) + *T***_***RT***_(1) * *SR*

        ***B***_***QS***_**(*M*) = *peak***_***x***_**(*M*) – *T***_***QR***_ * *SR*

C. Process The Rest Cycles

        **for *n* = 2:*M* − 1**

            ***B***_***SE***_**(*n*) = *peak***_***x***_**(*n*) + *T***_***RS***_ * *SR*

            ***B***_***TE***_**(*n*) = *peak***_***x***_**(*n*) + *T***_***RT***_(*n*) * *SR*

            ***B***_***QS***_**(*n*) = *peak***_***x***_**(*n*) − *T***_***QR***_ * *SR*

        **end for**

#### Sequence reorganization

The four regions are further determined based on the boundaries derived from Algorithm 2. ECG fragments in each cardiac cycle are extracted and the same type of regions are juxtaposed together in order, as well as the head/tail regions of the ECG sequence are juxtaposed.

In summary, Algorithm 3 describes the overall process for adaptive region segmentation. This method is adaptive to the input personalized ECG signals in the following two aspects: the amplitude threshold for the R peak detection is automatically adjusted to fit the recent few R peaks; meanwhile, the end of the T wave, i.e., *B*_*TE*_, was determined by the experience time window automatically adjusted to the R-R interval of each cardiac cycle. Therefore, a common set of the ARSPL setup parameters could be adaptively used for personalized ECG synthesis without calibration.

**Algorithm 3: Adaptive Region Segmentation**

**Process:**

A. Signal Preprocessing

        Step 1: ECG detrending and denoising

B. Region Segmentation

        Step 2: Determining R peaks of ECG sequences

        Step 3: Deriving boundaries of each region

C. Sequence Reorganization

        Step 4: Splicing the head and tail region

        Step 5: Splicing other regions of the same type in the sequence

**Fig 4:**
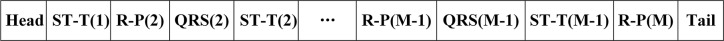
Adaptive region segmentation.

An illustration of adaptive region segmentation is shown in Fig 4. The resulting four subsequences for these four different regions are then used for linear regression training separately.

### Linear regression operation

After adaptive region segmentation, we obtain four reorganized subsequences, the ST-T, R-P, QRS, and H-T region sequences. Using the LR method expressed in (14), we derive four estimated matrices ***b***_***STT***_, ***b***_***RP***_, ***b***_***QRS***_, and ***b***_***HT***_ for the four regions. In contrast to the other three regions, the estimated matrix ***b***_***HT***_ for the H-T region is calculated using the complete ECG sequence rather than a region-based subsequence, as the head part is generally irrelevant to the tail part, and the region may contain many types of ECG sub-waves. To reconstruct standard 12-lead ECG sequences, adaptive region segmentation is applied to the sequences of the initial 3-lead subset for ECG synthesis. Based on (15), we reconstruct each region of the ECG signals using the four estimated matrices. Noticing that ARSPL is a personalized ECG synthesis method, subjects would go through their own model training and ECG synthesis process. That is, the four estimated matrices derived from an individual are only used to reconstruct the standard 12-lead ECGs of him/herself.

### ECG sequence restoration

To restore a final ECG sequence from the four region subsequences, the lengths of each cardiac cycle’s ECG fragments in the four regions are required. They are calculated based on (16)–(18).

$${SegL}_{STT}(n)=B_{TE}(n)-B_{SE}(n)$$ (16)

$${SegL}_{RP}(n)=B_{QS}(n)-B_{TE}(n-1)$$ (17)

$${SegL}_{QRS}(n)=B_{SE}(n)-B_{QS}(n)$$ (18)

where *SegL*_*STT*_(*n*), *SegL*_*RP*_(*n*), and *SegL*_*QRS*_(*n*) are the length of *ST*-*T*(*n*), *R*-*P*(*n*), and *QRS*(*n*), respectively. Based on the segment lengths, the *ST*-*T*(*n*), *R*-*P*(*n*), and *QRS*(*n*) can be re-split in the synthesized region sequences, and then combined via the inverse operation to the segmentation. The detailed steps are listed in Algorithm 4.

**Algorithm 4: ECG Sequence Restoration**

**Symbol setting:**

***M***: total number of R peaks

***SegL***_***STT***_**(*n*)**: length of ST-T region in cycle n

***SegL***_***RP***_**(*n*)**: length of R-P region in cycle n

***SegL***_***QRS***_**(*n*)**: length of QRS region in cycle n

**Process:**

Record the Length of Every Region in Each ECG Cycle

        Step 1: derive ***SegL***_***STT***_**(*n*),*n* = 1,2,…,*M* − 1**

        Step 2: derive ***SegL***_***RP***_**(*n*),*n* = 2,3,…,*M***

        Step 3: derive ***SegL***_***QRS***_**(*n*),*n* = 2,3,…,*M* − 1**

B. Recover the ECG from the ST-T, R-P, and QRS Regions

        Step 4: split the ***ST*-*T*(1)** in ST-T region sequence according to ***SegL***_***STT***_**(1)** and paste it to the restored sequence

        Step 5: split the ***R*-*P*(*n*)**, ***QRS*(*n*)**, and ***ST*-*T*(*n*)** in region sequences according to segment lengths ***SegL***_***RP***_**(*n*)**, ***SegL***_***QRS***_**(*n*)**, and ***SegL***_***STT***_**(*n*),*n* = 2,3,…,*M* − 1** strictly in order, and paste them to the restored sequence

        Step 6: split the ***R*-*P*(*M*)** in R-P region sequence according to ***SegL***_***RP***_**(*M*)** and paste it to the restored sequence

C. Recover the ECG from the H-T Region

        Step 7: split the first ***B***_***SE***_**(1)** points in H-T region sequence and paste it to the head of the restored sequence

        Step 8: paste the rest points in H-T region sequence to the tail of the restored sequence

## Experiments and results

### Study population

The PTB diagnostic ECG database contains 549 standard 12-lead ECG records from 290 subjects [15]. The records were digitized at 1000 Hz with 16-bit resolution over ± 16 mV. Of 268 subjects with clear clinical summaries, we established a study population of 39 pairs of 2-min digital ECGs from 39 subjects 51 ± 15 years of age (25 males and 14 females) randomly chosen from every diagnostic class of the database in proportion of to the original distributions. As a result, 8 pairs of standard 12-lead ECG records were from healthy volunteers and 31 pairs of the records have ECG abnormalities.

As ECG signals are individually specific, we divided the study population into two subsets, S1 and S2, for training and testing respectively. The two different records from each same subject were assigned to S1 and S2, respectively. For all four methods evaluated, personalized models were trained using the S1 records and then used to reconstruct the individual ECG records of S2. In other words, personalization was applied during both model building and ECG synthesis for each of the methods to evaluate the performance.

### Experimental settings

#### ARSPL settings

The synthesis accuracy of ARSPL relies on precise ECG segmentation, which, here, depends principally on R peak detection using Algorithm 1. Therefore, to evaluate the method and define the optimal settings, we applied Algorithm 1 to S1 and S2 using different setup parameters, i.e., wavelet bases, initial amplitude thresholds, and experiment time windows. The true-positive (TP) case (a correctly detected R peak), the false negative (FN) case (a missed R peak), and the false positive (FP) case (a noise spike detected as an R peak) were calculated. In addition, the sensitivity (Se), positive predictive value (+P), and overall detection accuracy (Acc) were derived using (19)–(21), respectively.

Se=TP/(TP+FN)×100% (19)

+P=TP/(TP+FP)×100% (20)

Acc=TP/(TP+FP+FN)×100% (21)

As a result, for ECG preprocessing, an 8-level discrete wavelet transform is applied to the raw ECG signal of the study population. The wavelet function is set to ‘sym5’ with the soft thresholding method described in [25] to remove the baseline wandering and other noises. For adaptive region segmentation, the R peak detection sequence is reconstructed using the level 3–5 detail coefficients of the original ECG signal. The initial amplitude threshold is set to 30% of the maximum value of the first two seconds of the ECG sequence. After the first three peaks are determined, the amplitude threshold will be consistently updated to 50% of the average value of the most recent three registered peaks’ amplitude. According to [29]-[30], the boundary experienced time window ***T***_***QR***_, ***T***_***RS***_, and the adjustment coefficient ***a***_***RT***_ are set to 0.03, 0.04, and 0.37 respectively.

Table 1 shows the performance of R peak determination with the optimal setup parameters. Of the 11953 beats from S1 and S2, Algorithm 1 afforded an overall detection accuracy of 98.71%, with a sensitivity of 99.01% and a positive predictive value of 99.69%. Thus, most R peaks were properly extracted; the ECGs were reliably divided into regions regarding the CEA stages via adaptive region segmentation preceding piecewise ECG synthesis.

**Table 1:** Performance of the R peak determination.

Total (beats)	TP (beats)	FN (beats)	FP (beats)	Se (%)	+P (%)	Acc (%)
11953	11916	119	37	99.01	99.69	98.71

#### BP and GA-BP settings

We use the same settings as [22] for BP and GA-BP. The hidden layer of the BP network features 15 neurons with tansig transfer functions, and the output neurons are linear activators. The number of iterations used to train the BP network was set to 605. For the genetic algorithm, the population size was set to 10 with a 50% crossover probability, a 10% mutation probability, and 33 generations.

#### Algorithm runtime

To evaluate runtimes, we implemented the four methods using MATLAB R2012a running on CentOS. The test platform featured two Intel Xeon E5-2620 CPUs (6/12 cores/threads, 2.0/2.5 GHz base/turbo) and 64 G 1600-MHz DDR3 RAM (4 G RAM were available for a single thread). The BP method with multiple networks has been compared with the GA-BP method in [22]. Hence, we simplified our experiments by configuring the BP method with a single network. The common LR method, the BP method, and the GA-BP method were compared with the ARSPL method.

### Experimental results

The four methods were trained using S1 with a data length of 10s, 20s, 30s, 40s, 50s, and 60s respectively and tested using S2 to evaluate the performance. The synthesis quality was evaluated based on CC, RMSE, and ST-level CDR. The paired t-test [31] was used to compare differences between the ARSPL CC and RMSE and those of the other three algorithms. Additionally, algorithm complexity was evaluated based on runtime costs.

Table 2 demonstrates the average CC and RMSE results for V1 and V3 toV6 of all four methods with different training data lengths. To compare the results for different training data lengths, we use the least RMSE as the primary evaluation metric. For cases with the same RMSEs, we further compare their CCs. If the RMSEs and the CCs are still the same, we then consider the training data length. With this procedure, the best training data lengths for LR, BP, GA-BP, and ARSPL are 60s, 50s, 40s, and 50s respectively. Also, we define the basic case as the one using a 10-second data for training.

**Table 2:** Average CC and RMSE of the four methods.

Training Data Length (s)		10	20	30	40	50	60
**CC**	**LR**	0.872	0.892	0.913	0.918	0.917	0.919
	**BP**	0.878	0.892	0.901	0.895	0.903	0.900
	**GA-BP**	0.912	0.920	0.914	0.918	0.918	0.915
	**ARSPL**	0.944	0.946	0.947	0.948	0.948	0.948
**RMSE** **(μV)**	**LR**	82.3	74.9	70.2	66.1	66.4	65.4
	**BP**	87.8	77.0	74.8	75.5	73.1	75.2
	**GA-BP**	68.6	67.1	67.3	65.8	67.0	67.2
	**ARSPL**	56.5	55.7	55.3	55.1	54.9	54.9

For the basic case, the average CC result of ARSPL is 0.944, showing a significant improvement compared to LR, BP, and GA-BP, for which the results are 0.872, 0.878, and 0.912 respectively (p ≤ 0.01). For the best case, the average CC result of ARSPL is 0.948, while the results of LR, BP, and GA-BP are 0.919, 0.903, and 0.918 respectively. The differences are also significant (p ≤ 0.01). As for the RMSE, the average result of ARSPL for the basic case is 56.5 μV, while the results of LR, BP, and GA-BP are 82.3 μV, 87.8 μV, and 68.6 μV respectively. And for the best case, the average RMSE result of ARSPL is 54.9 μV, and they are 65.4 μV, 73.1 μV, and 65.8 μV for LR, BP, and GA-BP respectively. The advantages of ARSPL over LR, BP, and GA-BP are clear and significant for both cases (p ≤ 0.01).

**Fig 5:**
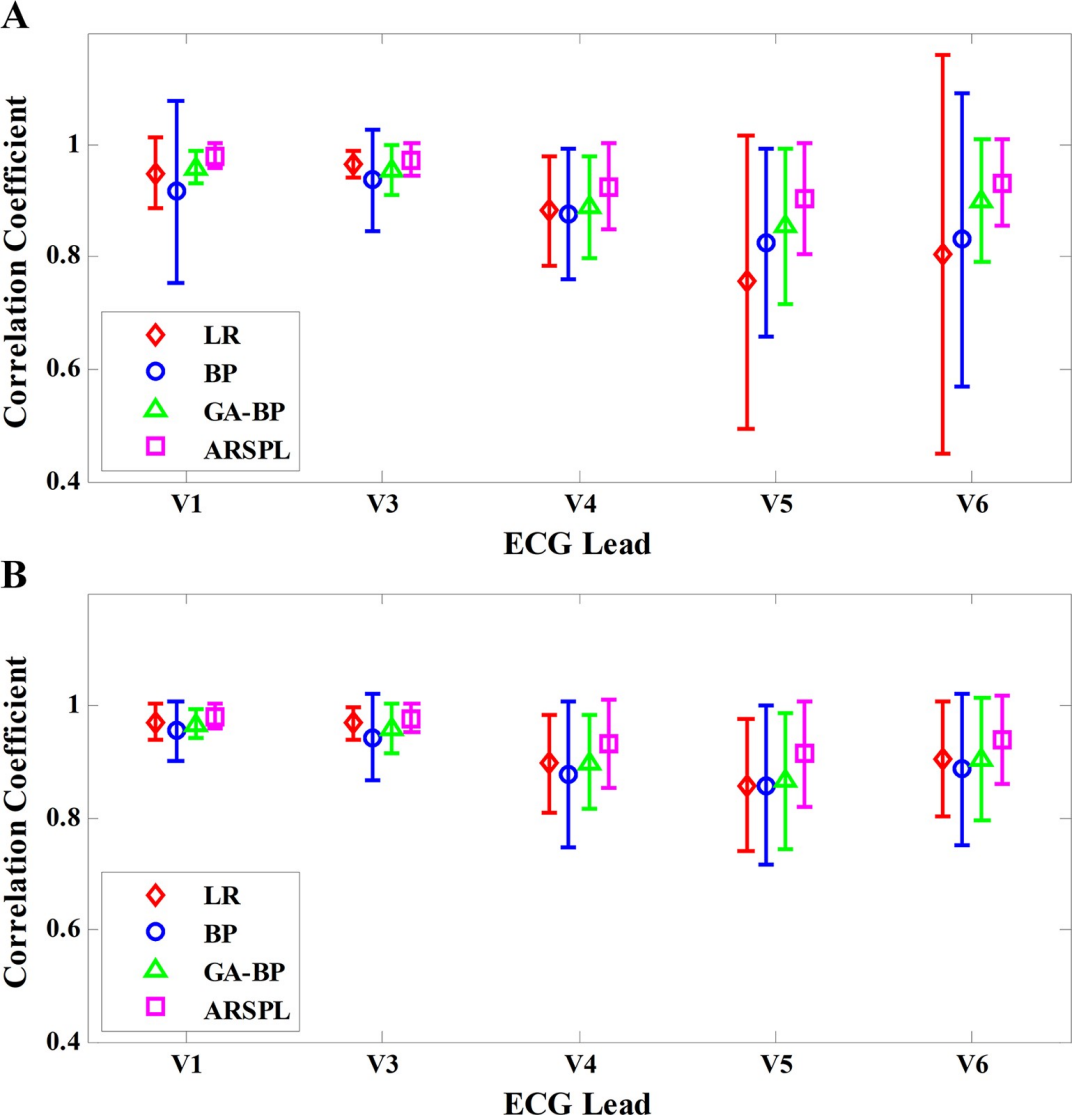
CC result of each lead for the basic/best cases. (A) CC comparison in the basic case. (B) CC comparison in the best case.

The detailed CC results of each lead of the four methods for the basic case and the best case are shown in Fig 5. For both cases, the ARSPL method has the best ECG reconstruction performance with respect to CC among the four methods for every lead. The differences are all significant (p ≤ 0.05), except Lead V3 compared to LR for both cases (p = 0.10 for the basic case and p = 0.08 for the best case), and Lead V6 compared to GA-BP (p = 0.07 for the basic case and p = 0.06 for the best case). The ARSPL method achieves a relatively high reconstruction accuracy for Leads V1 and V3, with a CC of 0.980/0.981 for Lead V1 and 0.974/0.977 for Lead V3 for the basic/best cases respectively, while Lead V5’s reconstruction accuracy is slightly lower, with a CC of 0.905/0.914 for the basic/best cases.

**Fig 6:**
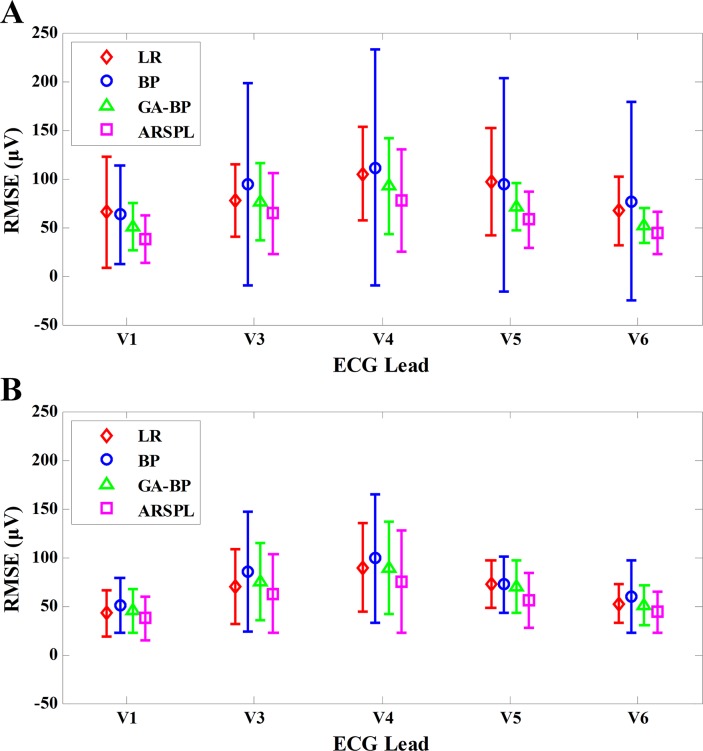
RMSE results of each lead for the basic/best cases. (A) RMSE comparison in the basic case. (B) RMSE comparison in the best case.

Fig 6 illustrates the detailed RMSE results of the four methods for each synthesis lead. As shown, for both cases, the ARSPL method achieves the lowest RMSE for every lead reconstruction among the four methods. Furthermore, the advantage of the ARSPL over the other three methods is significant (p ≤ 0.05) for the synthesis of Lead V1 for the basic case and Lead V5 for both cases. Also, ARSPL outperforms the LR and BP methods significantly (p ≤ 0.05) for Lead V6 reconstruction of both cases. However, the differences of RMSE between ARSPL and the other three methods are not significant for the synthesis of Leads V3 and V4, especially when comparing ARSPL with GA-BP, where the RMSE difference between them for Lead V6 is not significant either.

There could be discontinuities in amplitudes between regions of the ECGs synthesized by ARSPL. Table 3 lists the absolute means of the amplitude gaps between the R-P and QRS (G1), the QRS and ST-T (G2), and the ST-T and R-P regions (G3) in different leads for the basic/best cases. The average gaps in the three regions were all less than 40 μV. Moreover, the absolute means of all gaps were less than the RMSEs of the corresponding leads in both cases, indicating that these gaps were not the main cause of the synthesis error. Therefore, no specific technique was applied to deal with the amplitude gaps between regions in the ARSPL framework. This issue will be discussed later in the next section.

**Table 3:** Absolute means of the amplitude gaps.

Lead		V1	V3	V4	V5	V6	Average
**Basic Case (μV)**	**G1**	38.5	33.0	46.6	40.8	28.5	37.5
	**G2**	27.7	38.5	48.5	29.3	16.6	32.1
	**G3**	18.5	21.8	31.0	23.1	12.9	21.5
**Best Case (μV)**	**G1**	32.7	33.1	42.7	36.5	24.6	33.9
	**G2**	26.7	37.9	45.4	34.7	19.3	32.8
	**G3**	19.9	16.0	29.0	23.7	17.7	21.3

Denivelation of synthesized ECGs could affect the diagnosis of some indicator-sensitive pathological changes such as ST elevation. Therefore, for all four methods, we measured the ST-level as revealed by Lead V1 to further calculate the ST-level CDR, ER, and DR using (2)–(4). Table 4 presents the ST-level CDRs for both basic and best cases. The ARSPL method was associated with the lowest ST-level CDR: 12.74% for the basic case and 10.71% for the best case of 1727 cardiac cycles. In terms of the impact on the diagnosis of ST elevation, both the ER and the DR in critical denivelation situation of ARSPL were smaller than those of BP and GA-BP methods. Although the LR method was associated with the lowest DRs for both cases, ARSPL achieved much more lower ERs than those of LR for the basic/best cases, as more than 10%/7% lower ERs, respectively; whereas the DRs of ARSPL were only slightly higher compared to LR method.

**Table 4:** ST-level critical denivelation ratio.

Method		LR	BP	GA-BP	ARSPL
**Basic** **Case**	**CDR**	19.86%	16.74%	16.27%	12.74%
	**ER**	13.49%	4.17%	4.17%	3.13%
	**DR**	6.37%	12.57%	12.10%	9.61%
**Best** **Case**	**CDR**	17.03%	12.85%	15.41%	10.71%
	**ER**	10.89%	4.34%	3.71%	3.18%
	**DR**	6.14%	8.51%	11.70%	7.53%

**Fig 7:**
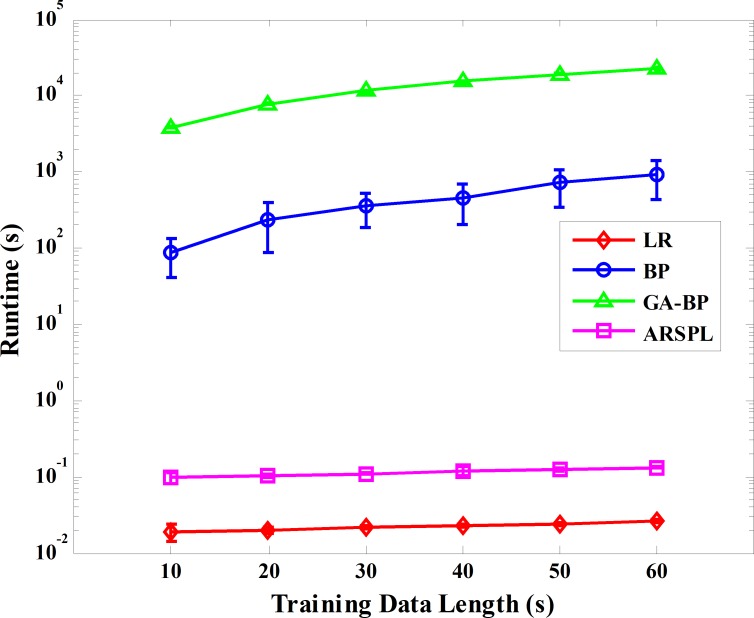
Algorithm runtime comparison.

Finally, in terms of time complexity, those of ARSPL and LR were *O*(*g*^2^ × *n*) [32], where *g* approximates the size of the linear coefficients and *n* the data size, and *n* ≫ *g*. For the BP and GA-BP methods, the time complexity was *O*(*w*^3^) for a single time step, where *w* approximates the parameter scale [33]-[34]. However, as the number of iterations required by the BP and GA-BP methods are not deterministic, averaging of actual runtimes rather than derivation of big O complexity is more appropriate. Fig 7 shows the algorithm runtimes, including both training and reconstruction times, of the four methods running on the above-mentioned platform. All four Matlab implementations automatically link to the Intel Math Kernel Library that contains highly optimized general matrix- matrix multiplication subroutines fully exploiting the parallelism and vectorization of the Intel Xeon processor [35]. Thus, the increase in the runtime with data length will differ from that derived by calculating big O complexity. Please note that the runtimes are presented in logarithmic coordinates. The runtimes of the ARSPL and LR methods increase only slowly with training data length, yet the BP and GA-BP runtimes increase significantly.

The detailed runtimes of the four methods for the basic and the best cases are listed in Table 5. The runtime of ARSPL is about 5 times longer than the LR method, while it is still in the order of a small fraction of a second. For common LR processes, the runtime would be shorter when processing the data as multiple sub-segments than processing the complete data at once, if the total amount of data is the same. So, the ARSPL method should be faster than the LR method benefitting from the segmentation operations. However, for the ARSPL method, the reconstruction of the H-T region needs the estimated matrix ***b***_***HT***_ trained from the complete ECG sequence, where the situation is the same as the LR method, thus the total amount of data processed by the ARSPL method is almost twice that of the LR method in the training process. As a result, the ARSPL method is only faster than the LR method in the synthesis process. Also, compared to the LR method, the ARSPL method spends more time for the adaptive region segmentation and signal reorganization processes, of which the time complexities are both *O*(*n*) [32]. In our experiment, the adaptive region segmentation, on average, accounts for 59.06% and 67.77% of the total training and synthesis time respectively, while the signal reorganization accounts for 13.59% of the total synthesis time. On the other hand, the BP and GA-BP methods took much more runtime, mainly during the training process, to reconstruct the standard 12-lead ECG signal, where the standard deviation (SD) of their runtimes are also relatively large, which reflects the randomness of the duration of training process.

**Table 5:** Runtime of the four methods.

Method		LR	BP	GA-BP	ARSPL
**Basic** **Case**	**Mean (s)**	0.019	86.3	3853	0.099
	**SD (s)**	0.005	44.7	71.0	0.016
**Best** **Case**	**Mean (s)**	0.026	711.4	15318	0.124
	**SD (s)**	0.001	364.6	320	0.004

The time costs of ECG reconstruction in the best case are listed in Table 6. Please note that the reconstruction time is irrelevant to the length of the training data, i.e., different cases. The ARSPL reconstruction time includes the time costs of adaptive region segmentation of test sequences, LR calculation, and sequence restoration. The time complexity of the ARSPL and LR ECG reconstruction is *O*(*g* × *n*) and *O*(*w* × *n*) for the BP and GA-BP methods [32]. Thus, when reconstructing a 2-min ECG sequence, the LR and ARSPL methods were slightly faster than the BP and GA-BP methods, principally because the parameter scale of the neural network was larger than those of the estimated matrices.

**Table 6:** Reconstruction time of the four methods in the best case.

Method	LR	BP	GA-BP	ARSPL
**Mean (s)**	0.017	0.111	0.111	0.086
**SD (s)**	0.002	0.003	0.003	0.005

It is expected that the advantage of the LR and ARSPL methods will become less significant as the length of the ECG sequence to be reconstructed increases. However, the reconstruction time among these methods are all trivial, especially taking into account the practical application scenarios, where the standard 12-lead ECGs are usually interpreted by the staff in the medical centers, and such reconstruction time is often negligible. So, for the actual use of ECG synthesis, the main impact of the runtime is about the training time, which will be discussed in the next section.

## Discussion

Based on the results and our findings, we briefly discuss four issues below.

### Segment-based ECG synthesis

The moving dipole model indicated that the performance of ECG synthesis will be improved based on segmented ECGs. As the heart vector changes with the CEA stage during the cardiac cycle, identifying and utilizing internal consistency within each stage allows model-building to converge more rapidly, which is equivalent to a need for less training data. The results confirm that adaptive region segmentation endowed the ARSPL method with the best synthesis accuracy and reduced sensitivity to training data length.

As segment-based ECG synthesis was accurate, adaptive region segmentation could also be used to enhance the non-linear methods. As suggested in [6] and [22], non-linear synthesis methods such as BP and GA-BP are superior to linear methods, especially when considering the effects of bodily factors on the relationships among lead signals over time. Therefore, if segmented ECGs were used for training, non-linear methods would afford better synthesis accuracy than would the ARSPL method. Nevertheless, the ARSPL method is designed to be accurate and efficient based on a linear method.

However, unlike methods based on complete ECG signals, segment-based ECG synthesis methods are associated with amplitude discontinuities between conjoint regions; the regional models were separately derived. In the ARSPL framework, these gaps are untreated, because the denivelations are small compared with the RMSE. However, spine- smoothing [36] and piecewise linear continuous functions [37] could be used to eliminate such discontinuities, improving segment-based ECG synthesis methods including ARSPL.

### Drawbacks and potentials of the ARSPL method

By introducing the H-T region and deploying simplified ECG segmentation, we ensured that the ARSPL framework was lightweight, and thus easily implemented from both the software and hardware perspectives; ARSPL is suited to mobile health systems. However, these advantages come with some cost in terms of accuracy.

As explained before, we used an H-T region to eliminate segmentation randomness, ensuring algorithmic simplicity. The data length of the H-T region can attain up to two cardiac cycles. If the ECG signal to be synthesized is relatively long, the H-T region can be ignored; there is no need to estimate ***b***_***HT***_ from the complete ECG sequence. Alternatively, the H-T region could be divided and subsumed in the other three regions prior to LR, further improving synthesis accuracy. In such a case, there is again no need the ***b***_***HT***_, yet the segmentation pattern (the type of the starting region of ECGs to be synthesized) is required during every ECG restoration.

The ARSPL method uses segmented ECGs to build reliable models by reference to changes in the heart vector. Therefore, precise ECG segmentation by CEA stage is key. An accurate R peak detection result could ensure that all beats of the ECG signals could be taken account for model training, and right ECG segments could be assigned to their corresponding ECG regions; thus those regions could distinguish different CEA stages correctly, enhancing ECG synthesis. Using optimal setup parameters, Algorithm 1 was 98.71% accurate in terms of R peak detection. Yet further improvement is possible, e.g., applying back-search strategy for potential missing peaks or using subsidiary criteria to eliminate interferences. Also, rather than using experienced time windows based on R peaks, region segmentation could exploit other existing feature points of ECGs, i.e., P peak, Q/S valley, T peak, etc., to obtain better matches between the regions and CEA stages at a cost of increased algorithmic complexity.

### Impact of the training time

Previous studies rarely considered the runtimes of synthesis algorithms, as training was usually performed offline, and trained algorithms were thus readily available for reconstructions, the runtimes of which were rather similar, as shown in Table 6. However, as individual-specific models are more accurate than generic models [6], reconstruction of individual standard 12-lead ECGs requires individualized training. Also training sets covering all possible normal and abnormal scenarios, especially in healthy individuals, are scarce. Thus, it is essential to update the model frequently for fast response to changes of the heart vector caused by heart disorders [38]; training runtime then becomes an issue.

The training process can be implemented either locally on a mobile device or remotely on a compute server. A local mobile device like a smart phone usually has limited computing resources, while a remote server requires the transmission of high-resolution ECG signals with a high sampling rate from the client to the server. Different from the LR and ARSPL methods, the BP and GA-BP methods require a long training time, about 10^2^ to 10^5^ seconds on a server, which makes it impractical for implementation on a local mobile device. Even if the training process is implemented on a remote server, the linear methods are still advantageous over the non-linear methods with respect to the training time, which is important for ECG synthesis scenarios requiring individualized models, frequent model updates, and quick responses. These advantages make the ARSPL method a much better candidate for either homecare with wearable devices and smart mobile devices or remote emergency medical care than the BP and GA-BP methods.

### Study limitations

In this study, the BP method featured a single neural network, for simplification. As discussed in [22], averaging of data from multiple neural networks improves synthesis performance. Our evaluation of the synthesis performance was based on test set S2, which consists of two-minute long ECGs from the PTB database. Although the ECGs in S1 and S2 were taken with an interval of 22 days on average from each subject, further experiments using longer ECGs with more pathologies are required to evaluate the consistencies of the ARSPL and other three methods. Moreover, the performance, e.g., latency and power consumption, of ARSPL on a real mobile platform or a simulated environment could be investigated.

## Conclusion

In this paper, we propose a novel method for standard 12-lead ECG synthesis from a 3-lead subset, i.e. I, II, and V2. Using adaptive region segmentation, our method performed significantly better than the common LR, BP, and GA-BP methods as revealed by CC, RMSE, and ST-level CDR. The method explores the change of the heart vector position within each cardiac cycle and retains the advantages of LR. Our lightweight method is especially suitable for remote emergency pre-diagnosis requiring a rapid response and for implementation in wearable ECG devices with limited computing resources. Synthesis is sensitive to training; a comprehensive set of normal and abnormal ECGs covering a wide range of individualized situations is required to further improve performance. Moreover, as ECG is used principally for cardiac diagnoses, synthesis methods combining the linear and nonlinear methods, i.e., using nonlinear methods for the reconstruction of ECG segments with key sensitive diagnostic characteristics in addition to ST-level, and linear methods for other parts of ECGs to reduce the overall training cost, could also be investigated.
